# Sensitivity of restriction spectrum imaging to memory and neuropathology in Alzheimer’s disease

**DOI:** 10.1186/s13195-017-0281-7

**Published:** 2017-08-02

**Authors:** Emilie T. Reas, Donald J. Hagler, Nathan S. White, Joshua M. Kuperman, Hauke Bartsch, Karalani Cross, Richard Q. Loi, Akshara R. Balachandra, M. J. Meloy, Christina E. Wierenga, Douglas Galasko, James B. Brewer, Anders M. Dale, Linda K. McEvoy

**Affiliations:** 1 Department of Radiology, University of California, San Diego, La Jolla, CA USA; 2 Center for Multimodal Imaging and Genetics, University of California, San Diego, La Jolla, CA USA; 3 Department of Psychiatry, University of California, San Diego, La Jolla, CA USA; 4 Wayne State University School of Medicine, Detroit, MI USA; 5 Department of Veterans Affairs, San Diego Healthcare system, La Jolla, CA USA; 6 Department of Neurosciences, University of California, San Diego, La Jolla, CA USA; 7 Department of Family Medicine and Public Health, University of California, San Diego, La Jolla, CA USA

**Keywords:** MRI, Diffusion imaging, Alzheimer’s disease, Memory, Mild cognitive impairment, Amyloid, Dementia

## Abstract

**Background:**

Diffusion imaging has demonstrated sensitivity to structural brain changes in Alzheimer’s disease (AD). However, there remains a need for a more complete characterization of microstructural alterations occurring at the earliest disease stages, and how these changes relate to underlying neuropathology. This study evaluated the sensitivity of restriction spectrum imaging (RSI), an advanced diffusion magnetic resonance imaging (MRI) technique, to microstructural brain changes in mild cognitive impairment (MCI) and AD.

**Methods:**

MRI and neuropsychological test data were acquired from 31 healthy controls, 12 individuals with MCI, and 13 individuals with mild AD, aged 63–93 years. Cerebrospinal fluid amyloid-β levels were measured in a subset (*n* = 38) of participants. RSI measures of neurite density (ND) and isotropic free water (IF) were computed in fiber tracts and in hippocampal and entorhinal cortex gray matter, respectively. Analyses evaluated whether these measures predicted memory performance, correlated with amyloid-β levels, and distinguished impaired individuals from controls. For comparison, analyses were repeated with standard diffusion tensor imaging (DTI) metrics of fractional anisotropy (FA) and mean diffusivity.

**Results:**

Both RSI and DTI measures correlated with episodic memory and disease severity. RSI, but not DTI, measures correlated with amyloid-β42 levels. ND and FA in the arcuate fasciculus and entorhinal cortex IF most strongly predicted recall performance. RSI measures of arcuate fasciculus ND and entorhinal cortex IF best discriminated memory impaired participants from healthy participants.

**Conclusions:**

RSI is highly sensitive to microstructural changes in the early stages of AD, and is associated with biochemical markers of AD pathology. Reduced ND in cortical association fibers and increased medial temporal lobe free-water diffusion predicted episodic memory, distinguished cognitively impaired from healthy individuals, and correlated with amyloid-β. Although further research is needed to assess the sensitivity of RSI to preclinical AD and disease progression, these results suggest that RSI may be a promising tool to better understand neuroanatomical changes in AD and their association with neuropathology.

**Electronic supplementary material:**

The online version of this article (doi:10.1186/s13195-017-0281-7) contains supplementary material, which is available to authorized users.

## Background

By the time of symptom onset in Alzheimer’s disease (AD), characteristic neuroanatomical changes have already begun to manifest. Although cognitive impairments are just emerging, cortical atrophy and white matter degeneration are detectable using magnetic resonance imaging (MRI) [1]. By the time these structural changes appear, the underlying neuropathology may render interventions to halt disease progression ineffective. Development of noninvasive tools to assess neural microstructure is critical to better characterize the earliest neurodegenerative events in AD, which in turn may permit the timely detection of incipient cognitive impairment and more effective intervention.

The earliest neuronal markers of AD include widespread amyloid-β (Aβ) deposits, and those that correspond most strongly with cognitive deficits appear in the medial temporal lobe of the brain, including neurofibrillary tangles, synapse loss, and neuronal death in the entorhinal cortex [2, 3]. Synapse loss, tau pathology, and cell death, together with white matter damage, spread throughout the limbic system and eventually to more extensive neocortical regions [4, 5]. Gray matter atrophy that mirrors progressive stages of this pathological cascade has demonstrated efficacy as an in-vivo marker of early AD. Although structural changes are widespread even at early disease stages [6], those in the medial temporal lobe, including entorhinal cortex and hippocampal atrophy, have the strongest associations with clinical and cognitive metrics [1, 7–11].

Diffusion MRI, of which the most widely used approach is diffusion tensor imaging (DTI), is based on the Brownian motion of water diffusion within biological tissue. DTI allows evaluation of neural microstructure that is complementary to standard morphometric MRI measures. Common DTI metrics include fractional anisotropy (FA; the magnitude of directed diffusion) and mean diffusivity (MD), which depend upon cellular barriers to water diffusion such as myelination, neuronal count, or number or density of neurites [12]. DTI studies have identified white matter changes co-occurring with or preceding gray matter atrophy in mild cognitive impairment (MCI) and AD [13]. White matter microstructure in the uncinate, superior longitudinal fasciculus, and fornix is altered in MCI and AD [14, 15] and predicts memory decline and progression from MCI to AD [16, 17]. Widespread white matter abnormalities in MCI and AD are at least partially independent of, and may precede, gray matter changes [18–20]. Within gray matter, diffusion imaging-based microstructural measures may be more sensitive than morphometry to disease onset and early neuropathology [21–23]. Diffusion neuroimaging may therefore have value for detecting preclinical neuropathological microstructural changes. However, studies have reported conflicting associations between FA and brain amyloid burden [24, 25], which may stem from the diversity of biological factors contributing to the aggregate diffusion tensor.

Restriction spectrum imaging (RSI) is a diffusion MRI method that enables measurement of microstructural features undetectable by conventional diffusion imaging techniques, and thus may permit earlier, perhaps presymptomatic, detection of incipient disease. Traditional DTI metrics inform about voxel-level features, but are blind to underlying subvoxel complexities, including crossing or bending fibers [26]. RSI resolves these properties by using multidirection, high b-value diffusion imaging to measure diffusion orientation and length scale [27]. RSI can consequently account for within-voxel crossing fibers and separate volume fractions of restricted, hindered, and free water diffusion [27], and may be less susceptible to the effects of edema or partial voluming than DTI. Histological examination in rodents has determined that the restricted volume fraction predominantly reflects intracellular diffusion within axons and dendrites, and is thus a valuable tool for probing gray and white matter neurite density (ND) [27]. RSI is clinically useful for tumor detection [28], and has characterized gray matter organization in autism [29] and white matter pathology in epilepsy [30].

This study examined the sensitivity of RSI metrics of ND and isotropic free water diffusion (IF) to memory impairment and disease status in MCI and AD. ND combines the volume fraction of mean restricted diffusion with the volume fraction of oriented diffusion that is highly restricted perpendicular to the direction of diffusion and is not attenuated by crossing fibers. ND therefore yields a combined measure of all restricted diffusion, which is likely dominated by neurites [27]. Although ND is strongly related to FA, FA is unable to separate restricted and hindered compartments or to account for crossing fibers, and can be reduced by partial voluming if the aggregate diffusion is isotropic. IF measures the volume fraction of isotropic free water diffusion, reflecting contributions from cerebrospinal fluid (CSF) and excluding hindered and restricted diffusion components. In comparison, MD measures average diffusion from all compartments.

ND and IF are expected to be sensitive to microstructural neural changes in MCI and AD, including white matter damage due to axonal degeneration or demyelination, and gray matter changes associated with atrophy or expansion of the CSF space. We therefore hypothesized that ND would be reduced and IF increased with greater dementia severity, episodic memory impairment, and Aβ burden, and that these metrics would accurately discriminate cognitively impaired patients from healthy controls (HC). For validation, RSI measures were compared against conventional DTI metrics.

## Methods

### Participants

Participants were recruited from the University of California, San Diego (UCSD) Shiley-Marcos Alzheimer’s Disease Research Center (ADRC). Participants completed standardized clinical evaluation through the ADRC Clinical Core, reviewed by two senior neurologists to provide a consensus diagnosis. AD diagnosis was based on NINCDS-ADRDA clinical criteria [31], and amnestic or multidomain MCI diagnosis was determined according to criteria outlined by Petersen et al. [32]. Exclusion criteria included a Mini-Mental State Examination score <16 indicating severe dementia, safety contraindications for MRI, uncorrected vision or hearing loss, significant illness, substance abuse, or major psychiatric or neurologic illness. HC were recruited from the ADRC and community. In addition to the above criteria, HC were excluded if they were taking psychotropic or cognitive enhancing medications, or demonstrated impairment on the Mattis Dementia Rating Scale (DRS) or Clinical Dementia Rating tests. After excluding three participants for poor diffusion imaging data quality, the final sample (*n* = 56; 30 women) included 31 HC, 12 participants with MCI, and 13 with AD, aged 63–93 years. CSF was obtained from a subset (68%; 24 HC, 7 MCI, 7 AD) of participants.

Study procedures were approved by the UCSD human subjects review board and participants provided informed, written consent prior to participation. Surrogate consent was provided for participants with advanced cognitive impairment.

### Neuropsychological assessment

The neuropsychological test battery, described previously [33], was administered by a trained examiner in a quiet room. Measures were selected for analysis based on their sensitivity to functional and memory impairments in AD. The DRS [34] assesses the nature and severity of dementia. The Functional Activities Questionnaire (FAQ) [35] assesses daily living activities. The WMS-R Logical Memory subtest [36] requires participants to report details of a passage, immediately and after delay. The California Verbal Learning Test (CVLT) [37] assesses the number of correctly recalled items from a list of 16 categorized words; immediate and delayed free recall were analyzed (CVLT-SFR and CVLT-LFR). The Consortium to Establish a Registry for Alzheimer’s Disease delayed recall (CERAD-DR) score [38] is another measure of verbal memory that tests delayed recall of a 10-item word list. The American National Reading Test Verbal IQ (ANART-VIQ) [39] was used as an estimate of premorbid ability.

### Imaging data acquisition and processing

MRI data acquisition was performed at the UCSD Center for functional MRI on a 3.0 tesla Discovery 750 scanner (GE Healthcare, Milwaukee, WI, USA) with an eight-channel phased array head coil. The MRI protocol included a three-plane localizer, a sagittal 3D fast spoiled gradient echo T_1_-weighted volume optimized for maximum gray/white matter contrast (TE = 3.2 ms, TR = 8.1 ms, inversion time = 600 ms, flip angle = 8°, FOV = 24 cm, frequency = 256, phase = 192, voxel size = 1 × 1 × 1.2 mm, scan time 8:27), and an axial 2D single-shot pulsed-field gradient spin-echo echo-planar imaging sequence (45-directions, b-values = 0, 500, 1500, 4000 s/mm2 and 1, 6, 6, 15 unique gradient directions for each b-value, respectively; TE = 80.6 ms, TR = 8 s, frequency = 96, phase = 96, voxel size = 1.875 × 1.875 × 2.5 mm, scan time 6:34).

Data were processed using an automated FreeSurfer-based processing stream (http://surfer.nmr.mgh.harvard.edu) with additional tools developed at the UCSD Multimodal Imaging Laboratory. Images were visually inspected for quality, and data containing motion or other artifacts were excluded from analysis. RSI data were corrected for motion and eddy current distortions [40], spatial and intensity distortions [41], and distortions caused by gradient nonlinearities [42]. Images were automatically registered and rigidly resampled into standard orientation, based on registration to T_1_-weighted structural images [43]. White matter tracts were labeled using a probabilistic atlas (AtlasTrack) [44] that combines information about fiber tract location and orientation to estimate the a posteriori probability that a voxel belongs to a tract of interest. To minimize partial volume effects, voxels containing primarily gray matter or CSF were excluded from the analysis of white matter tracts [45]. To correct for cortical surface partial volume effects, each voxel was assigned a volume fraction from 0–1 according to its proportion of gray or white matter. A weighting factor for each voxel was computed using Tukey’s bisquare weight function [46], setting volume fractions less than 0.5 to 0 and those above 0.5 to a weight between 0–1, to generate gray and white matter volume fraction maps. Gray matter, white matter, and CSF boundaries were delineated and cortical regions of interest were defined according to the Desikan-Killiany atlas [47]. DTI measures of FA and MD, and RSI measures of ND and IF were calculated within fibers and regions of interest. DTI measures were computed from all shells of the RSI acquisition using a nonlinear fitting procedure. Analysis of an independent dataset showed better correspondence to DTI measures derived from standard DTI data when using this method than a log transform followed by a linear fit.

### CSF collection and measurement

Lumbar puncture was performed by a board-certified neurologist, using a Sprotte atraumatic 24-gauge needle, between 8 am and 11 am after the participant had fasted overnight. Two milliliters of CSF were sent to a local laboratory for measurement of cell count, total protein, and glucose. The remainder (typically 15–20 ml) of CSF was gently mixed, centrifuged in a polypropylene conical tube at 1500 rpm for 10 min, then aliquotted into Sarstedt 0.5-ml cryotubes, snap-frozen immediately, and stored at –80°C until assayed. Levels of Aβ40 and Aβ42 were measured using mass spectrometry (Quest Diagnostics). CSF samples with gross blood contamination or with red blood cell counts >10/ml were not used.

### Data analysis

To minimize the number of fibers examined, analyses focused on tracts with connectivity to the temporal lobe that have previously demonstrated altered diffusion signal in MCI or AD [14, 15, 48]. FA and ND were measured in selected tracts, including fornix, parahippocampal cingulum, uncinate fasciculus, inferior longitudinal fasciculus (ILF), inferior fronto-occipital fasciculus (IFOF), and arcuate fasciculus. MD and IF of hippocampus and entorhinal cortex were assessed because of the critical role of these regions in memory and their vulnerability to degenerative changes in early AD.

Because there were no significant interactions between hemisphere and participant group for any RSI measure (all *p* > 0.01), values were averaged across hemispheres. Associations between diffusion imaging metrics and memory were assessed with partial correlations. Group differences in cognitive test scores, neuroimaging measures, and Aβ were evaluated using univariate general linear modeling (GLM). Post-hoc pair-wise group comparisons were adjusted for multiple comparisons using Bonferroni correction. Linear regression was conducted to identify neuroimaging metrics that predict cognitive function. To minimize the number of candidate variables and to allow comparison of RSI and DTI models, regression analyses were first performed separately for ND and IF, and for FA and MD. Significant predictors from these models were input as candidate variables into the final combined regression model for each neuropsychological measure. Linear discriminant analysis (LDA) was performed to distinguish HC from MCI/AD, and cross-validated classification accuracies were computed. LDAs were run separately for RSI metrics and for DTI metrics. Measures selected from these preliminary models were input into the combined LDA. Not all data met assumptions of normality and equal group covariances; however, LDA has been shown to be robust to data distribution and covariance violations [49]. Area under the receiver operating characteristic curve (AUC) was computed for each classifier, and AUCs were statistically compared [50]. RSI regression and LDA models were repeated with the inclusion of Aβ42 levels. Pearson’s correlations were calculated between memory and discriminant scores, and between Aβ levels and diffusion imaging metrics or memory scores. Significant differences between correlations were tested using Fisher r-to-z transformations.

For evaluation of whole brain group differences, voxel-based analysis was performed using the Advanced Normalization Tools (ANTS)-Groupwise processing pipeline, a modified Tract-Based Spatial Statistics (TBSS) processing pipeline (http://fsl.fmrib.ox.ac.uk/fsl/fslwiki/TBSS) which has improved algorithm accuracy and superior registration compared to standard TBSS [51, 52]. T_1_-weighted images were iteratively registered to form a groupwise map using the ANTS-Symmetric Normalization ver-1.9.4 algorithm [53]. Two-sample *t* tests (5000 general linear model permutations per contrast; http://fsl.fmrib.ox.ac.uk/fsl/fslwiki/Randomise/UserGuide) were performed on voxel-wise white matter ND and gray matter IF, contrasting MCI versus HC and AD versus HC. Voxels showing significant group differences (*p* < 0.01, threshold-free cluster enhancement with family-wise error (FWE) correction) were overlaid on the groupwise structural map from all participants in each contrast.

Partial correlations, GLMs, regressions, LDAs, and voxel-wise contrasts were adjusted for age, sex, and education. *P* < 0.01 was considered statistically significant. Data were analyzed using SPSS version 23.0 (IBM Corp, Armonk, NY, USA).

## Results

### Participant characteristics

HC, MCI, and AD groups did not differ in terms of age (*p* = 0.43) or education (*p* = 0.07), but MCI and AD groups had a higher proportion of men than HC (*p* = 0.007) (Table 1). After adjustment for age, sex, and education, there was a trend for higher levels of CSF Aβ42 for HC than MCI or AD (*F*(2,32) = 4.58, *p* = 0.02), whereas Aβ40 levels did not differ between groups (*p* = 0.31).

**Table 1:** Demographics, Aβ and neuropsychological test scores for each participant group

	HC (*n* = 31)	MCI (*n* = 12)	AD (*n* = 13)	Group effect
Age, years (range)	75.7 ± 5.6 (65–87)	77.4 ± 9.3 (63–93)	78.6 ± 7.8 (64–91)	*F*(2,53) = 0.85; *p* = 0.43
Sex (% women)	71%	33%	31%	x^2^ = 8.47; *p* = 0.007
Education, years (range)	15.8 ± 2.4 (8–20)	17.7 ± 2.0 (14–20)	16.0 ± 2.6 (12–20)	*F*(2,53) = 2.85; *p* = 0.07
DRS	139.8 ± 1.2	131.7 ± 2.0^a^	120.7 ± 1.9^b,c^	*F*(2,50) = 34.22; *p* < 0.001
FAQ	0.8 ± 0.8	3.0 ± 1.4	14.6 ± 1.1^b,c^	*F*(2,45) = 49.19; *p* < 0.001
LMI	15.6 ± 1.0	8.7 ± 1.5^b^	5.4 ± 1.4^b^	*F*(2,49) = 18.31; *p* < 0.001
LMD	14.2 ± 1.1	5.2 ± 1.7^b^	1.9 ± 1.6^b^	*F*(2,49) = 22.20; *p* < 0.001
CVLT-SFR	10.4 ± 0.5	2.9 ± 0.8^b^	1.9 ± 0.8^b^	*F*(2,45) = 43.36; *p* < 0.001
CVLT-LFR	11.1 ± 0.4	3.2 ± 0.7^b^	1.5 ± 0.7^b^	*F*(2,45) = 78.42; *p* < 0.001
CERAD-DR	7.2 ± 0.4	2.8 ± 0.6^b^	1.0 ± 0.6^b^	*F*(2,48) = 38.82; *p* < 0.001
ANART-VIQ	118.3 ± 1.4	117.4 ± 2.2	115.1 ± 2.0	*F*(2,46) = 0.85; *p* = 0.43
	*n* = 24	*n* = 7	*n* = 7	
Aβ40, ng/ml	15.8 ± 1.2	11.5 ± 2.5	16.1 ± 2.3	*F*(2,32) = 1.21; *p* = 0.31
Aβ42, ng/ml	3.0 ± 0.2	1.5 ± 0.5	2.0 ± 0.4	*F*(2,32) = 4.58; *p* = 0.02

Aβ and test scores are corrected for age, sex and education

Values are shown as mean ± standard error unless otherwise noted

^a^ *p* < 0.01, compared to HC; ^b^ *p* < 0.001, compared to HC; ^c^ *p* < 0.001, compared to MCI, with Bonferroni correction for multiple comparisons

*Aβ* amyloid-β, *AD* Alzheimer’s disease, *ANART-VIQ*, American National Reading Test Verbal IQ, *CERAD-DR* Consortium to Establish a Registry for Alzheimer’s Disease delayed recall, *CVLT-LFR* California Verbal Learning Test long delay free recall, *CVLT-SFR* California Verbal Learning Test short delay free recall, *DRS* Dementia Rating Scale, *FAQ* Functional Assessment Questionnaire, *HC* healthy controls, *LMI* Logical Memory immediate, *LMD* Logical Memory delayed, *MCI* mild cognitive impairment

As expected, significant group differences (adjusted for age, sex, and education) were observed on measures of dementia severity, functional ability, and memory (all *p* < 0.001), but not on the ANART-VIQ (*p* = 0.43) (Table 1). Pairwise comparisons revealed lower DRS (*p* < 0.01), Logical Memory immediate (LMI) and delayed (LMD) recall, CVLT short (CVLT-SFR) and long (CVLT-LFR) delay free recall, and CERAD-DR (all *p* < 0.001) scores for individuals with MCI compared to HC. Participants with AD scored worse than HC on the DRS, FAQ, and all memory measures, and worse than participants with MCI on the DRS and FAQ (all *p* < 0.001).

### Associations between diffusion imaging metrics and memory

Partial correlations of RSI measures with memory measures, adjusted for age, sex, and education, are shown in Table 2. CVLT-SFR positively correlated with IFOF and arcuate fasciculus ND, and CVLT-LFR positively correlated with fornix, uncinate, IFOF, and arcuate ND. CERAD-DR positively correlated with ND in the fornix, uncinate, ILF, IFOF, and arcuate. All memory measures negatively correlated with entorhinal cortex IF, and LMI recall and CERAD-DR negatively correlated with hippocampus IF (all *p* < 0.01). No significant associations with memory were observed for ND in the parahippocampal cingulum. Correlations of memory scores with DTI measures are presented in Table 2. Correlations did not significantly differ between RSI and DTI measures (all *p* > 0.01).

**Table 2:** Partial correlations (*r*) between memory test scores and RSI and DTI measures

	LMI	LMD	CVLT-SFR	CVLT-LFR	CERAD-DR
RSI					
Fornix ND	0.30	0.35	0.38	0.42*	0.55**
Parahippocampal cingulum ND	0.15	0.13	0.14	0.15	0.32
Uncinate ND	0.10	0.06	0.37	0.41*	0.43*
ILF ND	0.07	0.21	0.34	0.37	0.43*
IFOF ND	0.22	0.37	0.43*	0.45*	0.61**
Arcuate ND	0.14	0.35	0.44*	0.43*	0.51**
Entorhinal IF	–0.39*	–0.50**	–0.43*	–0.57**	–0.64**
Hippocampus IF	–0.39*	–0.30	–0.38	–0.38	–0.47*
DTI					
Fornix FA	0.39*	0.28	0.31	0.36	0.51**
Parahippocampal Cingulum FA	0.19	0.10	0.04	0.04	0.29
Uncinate FA	0.06	0.15	0.48*	0.50**	0.51**
ILF FA	0.14	0.26	0.27	0.29	0.39
IFOF FA	0.39	0.40*	0.38	0.41*	0.60**
Arcuate FA	0.22	0.23	0.30	0.29	0.34
Entorhinal MD	–0.29	–0.41*	–0.35	–0.46*	–0.55**
Hippocampus MD	–0.23*	–0.27	–0.42*	–0.43*	–0.49**

Correlations corrected for age, sex, and education

*p < 0.01, **p < 0.001

*CERAD-DR* Consortium to Establish a Registry for Alzheimer’s Disease delayed recall, *CVLT-LFR* California Verbal Learning Test long delay free recall, *CVLT-SFR* California Verbal Learning Test short delay free recall, *DTI* diffusion tensor imaging, *FA* fractional anisotropy, *IF* isotropic free water diffusion, *IFOF* inferior fronto-occipital fasciculus, *ILF* inferior longitudinal fasciculus, *LMI* Logical Memory immediate, *LMD* Logical Memory delayed, *MD* mean diffusivity, *ND* neurite density, *RSI* restriction spectrum imaging

### Sensitivity of diffusion imaging metrics to MCI and AD

**Fig. 1:**
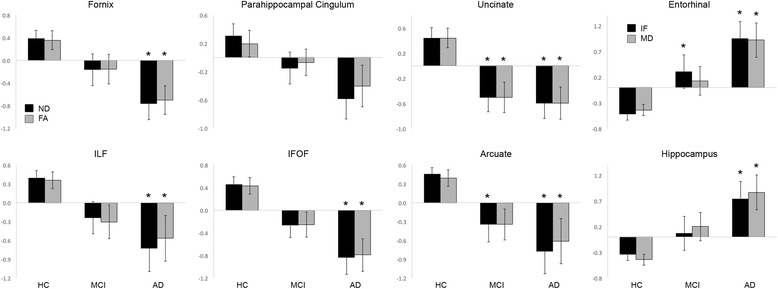
Group effects of RSI and DTI metrics. Standardized mean (± standard error) ND and IF (*black bars*) and FA and MD (*gray bars*) values, adjusted for age, sex, and education, are plotted for HC, MCI, and AD. *p < 0.01, compared to HC with Bonferroni correction for multiple comparisons. *AD* Alzheimer’s disease, *FA* fractional anisotropy, HC healthy controls, *IF* isotropic free water diffusion, *IFOF* inferior fronto-occipital fasciculus, *ILF* inferior longitudinal fasciculus, *MCI* mild cognitive impairment, *MD* mean diffusivity

Standardized group mean ND and IF values for each region and fiber of interest, adjusted for age, sex, and education, are plotted in Fig. 1. Significant main effects of group were observed for ND in the fornix (*F*(2,50) = 9.23, *p* < 0.001), uncinate (*F*(2,50) = 11.03, *p* < 0.001), ILF (*F*(2,50) = 9.05, *p* < 0.001), IFOF (*F*(2,50) = 13.41, *p* < 0.001), and arcuate (*F*(2,50) = 13.13, *p* < 0.001), and for IF in the entorhinal cortex (*F*(2,50) = 21.38, *p* < 0.001) and hippocampus (*F*(2,50) = 7.35, *p* = 0.002). ND values were lower for MCI than HC in the uncinate and arcuate, and lower for AD than HC in the fornix, uncinate, ILF, IFOF, and arcuate (all *p* < 0.01). IF in the hippocampus was higher for AD than HC, and IF in the entorhinal cortex was higher for MCI and AD than HC (all *p* < 0.01). No significant differences between MCI and AD were observed for any measure. ND in the parahippocampal cingulum did not differ between groups (*p* = 0.01).

For comparison, group effects for FA and MD within the same fibers and regions of interest as ND and IF are presented in Fig. 1. Although RSI and DTI effects were similar, there was a trend for stronger group effects for all ND measures than FA measures, and for entorhinal cortex IF than MD. Additional file 1 (Table S1) presents group effect sizes for RSI and DTI metrics.

**Fig. 2:**
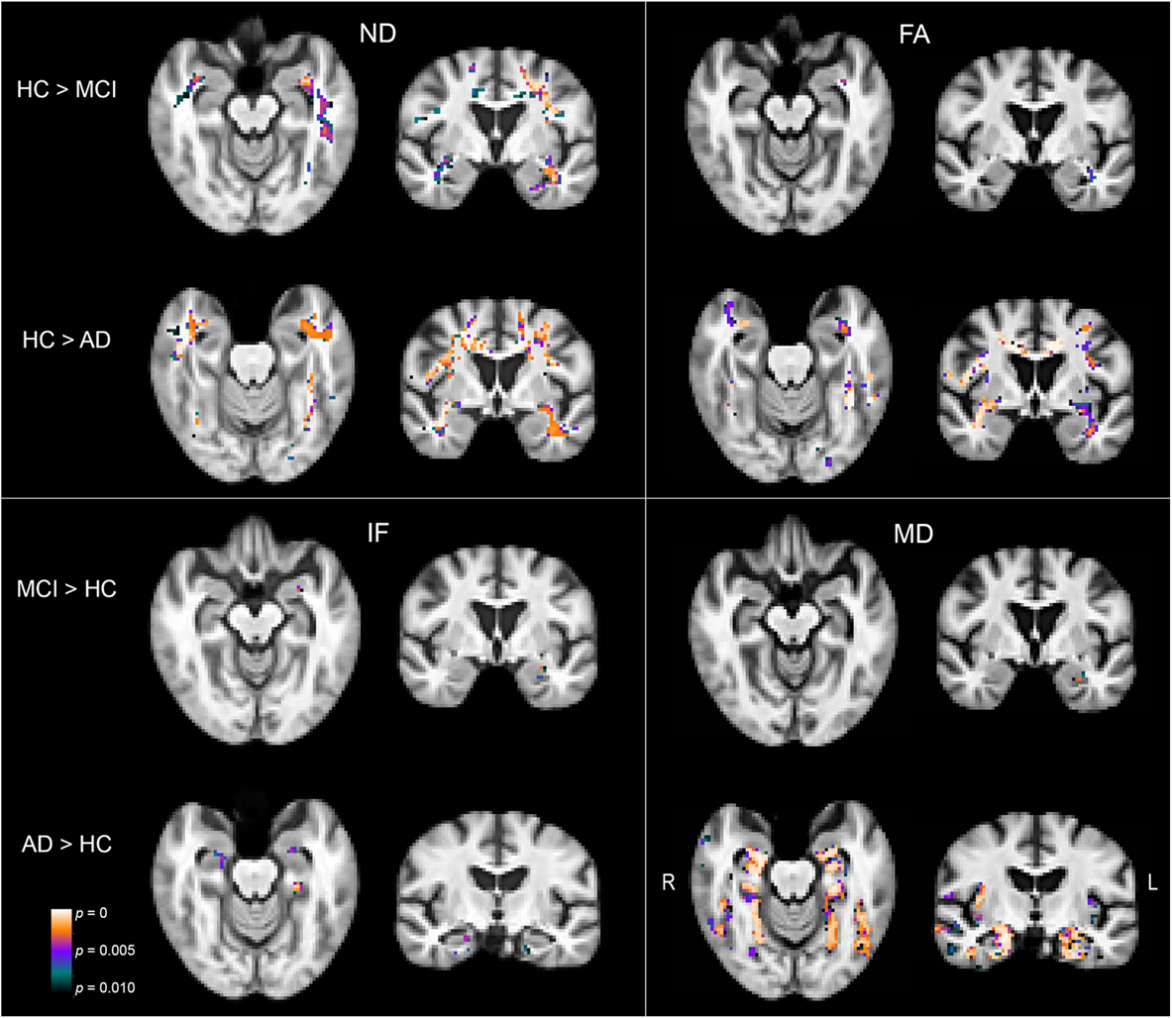
Whole-brain group differences in RSI and DTI metrics. Contrast maps show voxels with significantly lower ND or FA, and greater IF or MD, for MCI versus HC and for AD versus HC (*p* < 0.01, FWE cluster corrected). *AD* Alzheimer’s disease, *FA* fractional anisotropy, HC healthy controls, *IF* isotropic free water diffusion, *MCI* mild cognitive impairment, *MD* mean diffusivity

To visualize whole brain group differences, contrast maps compared voxel-wise ND and IF values for MCI versus HC and for AD versus HC (*p* < 0.01, FWE cluster corrected) (Fig. 2). Reduced white matter ND was observed bilaterally throughout the brain in AD and MCI compared to HC, although these differences had a more limited distribution in MCI. IF was increased in AD versus HC in the bilateral medial temporal lobe, whereas only one cluster in the left anterior medial temporal lobe showed increased IF in MCI versus HC. Voxel-wise differences in FA and MD are also shown in Fig. 2 for comparison. There were more extensive reductions in ND than in FA for both MCI and AD participants. There were more widespread increases in MD than IF for AD participants.

### Diffusion imaging predictors of cognitive function

Regression models to predict DRS and memory scores were run separately using RSI and DTI measures. There was a trend for RSI-based models to better predict each neuropsychological measure than DTI-based models (Additional file 1: Table S2). Regression models combining RSI and DTI measures are presented in Table 3. Only RSI measures predicted DRS (arcuate ND, hippocampus IF) and CVLT-SFR and CVLT-LFR (entorhinal IF, arcuate ND) scores. A combination of RSI and DTI measures predicted LMI and LMD recall (entorhinal IF, arcuate FA) and CERAD-DR (entorhinal IF, IFOF FA, fornix FA) scores.

**Table 3:** Beta values for predictors of neuropsychological test scores, with model *F* and *R* ^2^ values using RSI and DTI metrics

	Regressor	Beta	*F* value*	*R* ^2^
DRS	Arcuate ND Hippocampus IF	0.64 –0.31	16.01	0.62
LMI	Entorhinal IF Arcuate FA	–0.53 0.27	6.64	0.64
LMD	Entorhinal IF Arcuate FA	–0.61 0.23	8.74	0.69
CVLT- SFR	Entorhinal IF Arcuate ND	–0.45 0.34	11.47	0.75
CVLT- LFR	Entorhinal IF Arcuate ND	–0.58 0.28	16.63	0.81
CERAD - DR	Entorhinal IF IFOF FA Fornix FA	–0.49 0.33 0.25	16.44	0.82

Values corrected for age, sex, and education

*All regression models significant at *p* < 0.001

*CERAD-DR* Consortium to Establish a Registry for Alzheimer’s Disease delayed recall, *CVLT-LFR* California Verbal Learning Test long delay free recall, *CVLT-SFR* California Verbal Learning Test short delay free recall, *DRS* Dementia Rating Scale, *DTI* diffusion tensor imaging, *FA* fractional anisotropy, *IF* isotropic free water diffusion, *IFOF* inferior fronto-occipital fasciculus, *LMI* Logical Memory immediate, *LMD* Logical Memory delayed, *ND* neurite density, *RSI* restriction spectrum imaging

### Group classification

Because no differences were observed between MCI and AD on any RSI or DTI measure, these groups were combined into an “impaired” group for classification versus HC. Using RSI measures, entorhinal cortex IF and arcuate ND best distinguished HC from impaired participants (Wilks’ lambda = 0.58, x^2^ = 29.0 *p* < 0.001; 80% cross-validated classification accuracy). Using DTI measures, uncinate and arcuate FA and entorhinal cortex MD were selected (Wilks’ lambda = 0.60, x^2^ = 27.2 *p* < 0.001; 73% cross-validated classification accuracy). AUCs did not statistically differ (*p* = 0.83) between RSI (AUC = 0.89) and DTI (AUC = 0.88) classifiers. When the model selected from measures included in these RSI and DTI models, only RSI measures were chosen.

Scores for the DTI (all *p* < 0.01) and RSI (all *p* < 0.001) discriminant functions significantly correlated with all neuropsychological test scores (Table 4). Correlations for the RSI and DTI functions did not significantly differ. Additional file 1 (Figure S1) presents discriminant scores plotted against DRS scores.

**Table 4:** Pearson’s correlations (*r*) between discriminant scores and neuropsychological test scores

	DTI function	RSI function
DRS	0.65**	0.72**
LMI	0.48*	0.59**
LMD	0.52**	0.62**
CVLT- SFR	0.55**	0.62**
CVLT- LFR	0.54**	0.65**
CERAD - DR	0.67**	0.73**

*p < 0.01, **p < 0.001

*CERAD-DR* Consortium to Establish a Registry for Alzheimer’s Disease delayed recall, *CVLT-LFR* California Verbal Learning Test long delay free recall, *CVLT-SFR* California Verbal Learning Test short delay free recall, *DRS* Dementia Rating Scale, *DTI* diffusion tensor imaging, *LMI* Logical Memory immediate, *LMD* Logical Memory delayed, *RSI* restriction spectrum imaging

### Association of Aβ with memory and diffusion imaging metrics

**Fig. 3:**
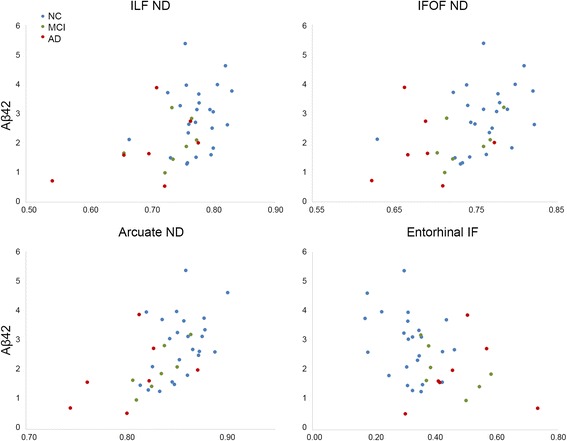
Association RSI measures and Aβ42 levels. ND and IF are plotted against Aβ42 levels (ng/ml). Values are shown for HC (*blue*), MCI (*green*), and AD (*red*) participants. *Aβ* amyloid-β, *AD* Alzheimer’s disease, HC healthy controls, *IF* isotropic free water diffusion, *IFOF* inferior fronto-occipital fasciculus, *ILF* inferior longitudinal fasciculus, *MCI* mild cognitive impairment, *MD* mean diffusivity, *ND* neurite density

Aβ42 levels correlated with CERAD scores (*r* = 0.43, *p* = 0.009) and there were trends for correlations with LMD recall (*r* = 0.33, *p* = 0.04) and CVLT-LFR (*r* = 0.38, *p* = 0.02). Aβ42 levels positively correlated with ND in the ILF (*r* = 0.44, *p* = 0.006), IFOF (*r* = 0.44, *p* = 0.006), and arcuate (*r* = 0.55, *p* < 0.001), and negatively correlated with entorhinal cortex IF (*r* = –0.42, *p* = 0.009) (Fig. 3). In contrast, Aβ42 did not correlate with FA or MD in any region examined (all *p* > 0.01). Aβ40 levels did not correlate with memory scores (all *p* > 0.30) or with any RSI or DTI measure (all *p* > 0.01).

When Aβ42 was added to RSI-based regression models, it was not included as a predictor of any memory score. Similarly, when Aβ42 was added to RSI-based LDA models, it was not selected to discriminate HC from impaired participants.

## Discussion

This study evaluated RSI metrics for sensitivity to disease status and cognitive deficits in MCI and AD. RSI-based measures of gray and white matter microstructure correlated with disease severity, functional ability, memory, and Aβ load.

White matter integrity of several tracts with temporal lobe projections significantly correlated with memory and distinguished impaired from healthy participants. These findings are consistent with prior reports that reduced FA in these pathways distinguishes MCI or AD from normal aging [14, 48], and tracks [15] or predicts [17, 54] disease progression. Effects were strongest for the IFOF, uncinate, and arcuate, long-range association fibers that connect temporal with frontal, occipital, and parietal cortex and may support integrative processing critical to memory or other cognitive functions that decline with disease progression.

Entorhinal cortex IF and MD were the strongest correlates of memory impairment and disease status, in line with the origins of AD neuropathology in the entorhinal area [2, 3]. Thus, this notable sensitivity of entorhinal cortex microstructural changes may reflect more advanced pathology in this focal region with relative sparing of other regions at the mild disease stages examined here. Entorhinal cortex but not hippocampal IF correlated with Aβ42, and increased free water diffusion has also been observed in the hippocampus but not entorhinal cortex in normal aging, highlighting the possible specificity of entorhinal microstructural change to prodromal AD [55].

RSI and DTI metrics are highly correlated, as demonstrated by their comparable associations with memory. However, we found preliminary evidence that RSI may offer additional information on microstructural changes to neural tissue and their association to early AD neuropathology. Intriguingly, lower ND and higher IF were respectively associated with greater burden (lower CSF levels) of Aβ42, the principal component of amyloid plaques and considered a crucial peptide for AD pathogenesis. In contrast, neither FA nor MD in any region correlated with Aβ levels, suggesting that RSI tracks neuropathological burden more closely than DTI. Prior studies report conflicting associations of Aβ with increased [25] or decreased [24] FA. These inconsistencies in the literature, and the lack of association of Aβ with FA or MD in our data, may derive from limitations of DTI to resolve microarchitectural complexities such as crossing fibers. Thus, RSI may provide the advantage over conventional diffusion imaging techniques of more complete characterization of tissue microstructure which may better inform about the relationship between tissue disorganization and its underlying pathophysiology.

Furthermore, there was a trend for stronger group differences for ND than FA in all fiber tracts, and for entorhinal cortex IF than MD, and discriminant analysis selected RSI over DTI metrics to distinguish impaired from healthy participants. When both RSI and DTI metrics were submitted to regression models, only RSI measures predicted dementia severity and CVLT scores, whereas no cognitive measure was predicted by DTI measures only. RSI and DTI measures differentially related to delayed recall, with a stronger association of arcuate ND to CVLT scores and of IFOF and fornix FA to CERAD scores (entorhinal cortex IF predicted both). The CVLT may be more sensitive to subtle memory impairments than the CERAD [56] and, here, delayed recall deficits for impaired individuals were also more severe for the CVLT than the CERAD. Microstructural changes in entorhinal cortex and arcuate, detectable with RSI, may therefore be powerful indices of mild memory impairment.

Although other advanced diffusion MRI techniques may also improve sensitivity to neuropathological tissue microarchitecture compared to DTI, each approach is distinct in its implementation efficiency and characterization of complex fiber geometry. RSI is a multishell, multicompartment extension of traditional high-angular resolution diffusion imaging (HARDI). While HARDI can resolve complex fiber orientations [57] it is blind to length scale information and thus cannot distinguish hindered and restricted diffusion pools. Diffusion kurtosis imaging (DKI) [58], which also employs a multishell, multidirection acquisition, only indirectly estimates the structural complexity of tissue from measures of diffusional kurtosis. Neurite orientation dispersion and density imaging (NODDI) [59], which like RSI integrates a multishell HARDI acquisition with a multicompartment model, can also separate tissue compartments and assess complex tissue microstructure. However, whereas NODDI characterizes the degree of fiber dispersion, RSI further identifies the geometric pattern of dispersion (e.g., crossing fibers) within a more efficient acquisition time (6.5 versus 30 min [59]). Our findings add to a small but growing literature demonstrating sensitivity of advanced diffusion imaging techniques such as DKI and NODDI to neural microstructural changes in AD. RSI may provide metrics of tissue architecture complementary to these approaches, to offer a more complete characterization of pathological brain microstructure in AD.

While further study is needed to identify the neurobiological substrates underlying reduced ND and increased IF in MCI and AD, these changes broadly reflect fewer barriers to diffusion within brain tissue. Prior histological validation indicates that ND correlates highly with neurite integrity [27], and reduced ND may arise from various factors including reduced axon or dendrite count or density, demyelination, or synapse loss [12, 60]. Greater isotropic free water diffusion in neurodegenerative disease could reflect expansion of the extracellular space related to neuronal loss, cell shrinkage, or tissue disorganization. Because RSI can isolate isotropic free water diffusion from restricted and hindered diffusion compartments, IF provides a more specific measure than average voxel diffusion, which may explain the more limited spatial distribution of AD-related changes observed for IF than MD in whole-brain analyses. Additional histological comparison and integration of RSI with multimodal imaging and computational modeling [61] may better clarify how microstructural brain changes relate to underlying cell pathology and brain network reorganization.

Although RSI overcomes many obstacles posed by conventional diffusion imaging methods, some remaining limitations warrant consideration. Partial volume effects may artificially deflate estimates of both FA and ND, although we attempted to account for partial voluming in the cortical surface and when identifying fiber tracts. These artifacts would be most problematic for fine tracts adjacent to CSF, such as the fornix [62]; here, the strongest predictors of cognitive function and disease were from thicker long-range association fibers that should be more robust to partial volume effects [63]. Clinical diagnoses were made according to standard criteria; nevertheless, MCI is a heterogeneous condition and even a diagnosis of AD is not definitive without postmortem validation. The stepwise increases in dementia severity and functional ratings from HC to MCI to AD suggest that these groups represent a spectrum from healthy to mildly impaired. However, because we enrolled only mildly impaired AD participants, and MCI and AD participants did not differ on measures of brain microstructure or memory, clinical overlap may exist between these groups. Because neural microstructure may not change linearly with disease progression, nonlinear associations of RSI metrics with severity of cognitive impairment will be a topic for follow-up investigation. Finally, diffusion imaging cannot directly inform about the cellular pathology mediating neuroanatomical differences. Future studies are needed to further assess the biological underpinnings of RSI biomarkers and evaluate their sensitivity to preclinical changes indicative of subsequent cognitive and functional decline.

## Conclusions

This study identified novel diffusion imaging markers of microstructural changes in brain gray and white matter in MCI and AD. Reduced neurite density in multiple white matter tracts and increased medial temporal lobe free water diffusion strongly associated with memory deficits, disease status, and pathophysiology. These findings suggest that RSI is highly sensitive to microstructural changes in the early stages of neurodegenerative memory disease, supporting its potential utility as an early biomarker of preclinical neuropathological events.

## Additional file

Additional file 1: Figure S1. Association between discriminant scores and DRS scores. Discriminant scores for DTI and RSI discriminant functions are plotted against DRS scores. Scores are shown for HC (*blue*), MCI (*green*), and AD (red) participants. **Table S1.** Group effect sizes (partial eta-squared) for RSI (ND, IF) and DTI (FA, MD) measures. **Table S2.** Beta values for predictors of neuropsychological test scores, with model F and R^2^ values (corrected for age, sex, and education), using RSI metrics (A) and DTI metrics (B). (DOCX 78 kb)

## Abbreviations

Aβ: Amyloid-β
AD: Alzheimer’s disease
ANART-VIQ: American National Reading Test Verbal IQ
AUC: Area under the receiver operating characteristic curve
CERAD-DR: Consortium to Establish a Registry for Alzheimer’s Disease delayed recall
CSF: Cerebrospinal fluid
CVLT-LFR: California Verbal Learning Test long delay free recall
CVLT-SFR: California Verbal Learning Test short delay free recall
DKI: Diffusion kurtosis imaging
DRS: Dementia Rating Scale
DTI: Diffusion tensor imaging
FA: Fractional anisotropy
FAQ: Functional Activities Questionnaire
FWE: Family-wise error
GLM: General linear modelling
HARDI: High-angular resolution diffusion imaging
HC: Healthy controls
IF: Isotropic free water diffusion
IFOF: Inferior fronto-occipital fasciculus
ILF: Inferior longitudinal fasciculus
LDA: Linear discriminant analysis
LMD: Logical Memory delayed
LMI: Logical Memory immediate
MCI: Mild cognitive impairment
MD: Mean diffusivity
MRI: Magnetic resonance imaging
ND: Neurite density
NODDI: Neurite orientation dispersion and density imaging
RSI: Restriction spectrum imaging
