# Supplementary material for: Sensitivity of restriction spectrum imaging to memory and neuropathology in Alzheimer’s disease
Source: Alzheimers Res Ther. 2017 Aug 2;9:55. doi: 10.1186/s13195-017-0281-7 (PMC5539622; doi:10.1186/s13195-017-0281-7)
Supplement: Additional file 1: Figure S1. — Association between discriminant scores and DRS scores. Discriminant scores for DTI and RSI discriminant functions are plotted against DRS scores. Scores are shown for HC (blue), MCI (green), and AD (red) participants. Table S1. Group effect sizes (partial eta-squared) for RSI (ND, IF) and DTI (FA, MD) measures. Table S2. Beta values for predictors of neuropsychological test scores, with model F and R2 values (corrected for age, sex, and education), using RSI metrics (A) and DTI metrics (B). (DOCX 78 kb) [file 13195_2017_281_MOESM1_ESM.docx]

**Additional file 1**

**
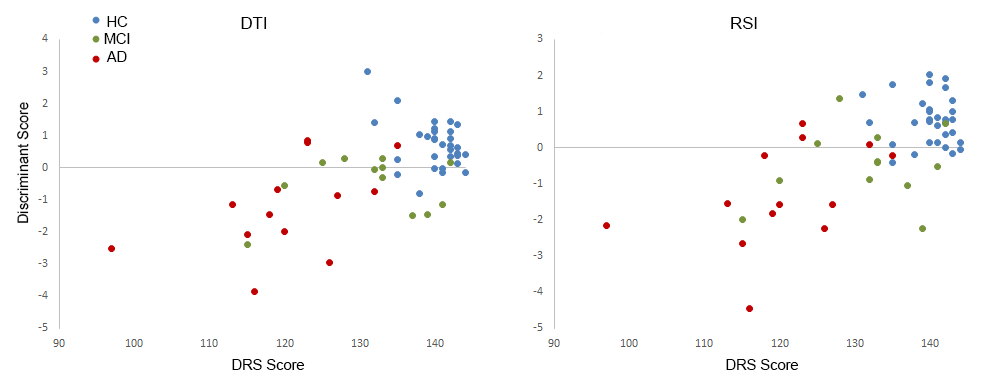
**

**Figure S1. Association between discriminant scores and DRS scores.** Discriminant scores for DTI and RSI discriminant functions are plotted against DRS scores. Scores are shown for HC (blue), MCI (green) and AD (red) participants.

**Table S1**. Group effect sizes (partial eta-squared) for RSI (ND, IF) and DTI (FA, MD) measures.

|  | **ND** | **IF** | **FA** | **MD** |
| --- | --- | --- | --- | --- |
| **Fornix** | 0.27 |  | 0.23 |  |
| **Parahippocampal Cingulum** | 0.16 |  | 0.07 |  |
| **Uncinate** | 0.31 |  | 0.31 |  |
| **ILF** | 0.27 |  | 0.20 |  |
| **IFOF** | 0.35 |  | 0.32 |  |
| **Arcuate** | 0.34 |  | 0.24 |  |
| **Entorhinal** |  | 0.46 |  | 0.36 |
| **Hippocampus** |  | 0.23 |  | 0.35 |

**Table S2**. Beta values for predictors of neuropsychological test scores, with model *F* and R² values (corrected for age, sex and education), using RSI metrics (A) and DTI metrics (B).

| **A. RSI Models** | **Regressor** | **Beta** | ***F*-value *** | **R²** |
| --- | --- | --- | --- | --- |
| **DRS** | Arcuate ND  Hippocampus IF | 0.64  -0.31 | 16.01 | 0.62 |
| **LMI** | Entorhinal IF  Arcuate ND  ILF ND | -0.55  0.75  -0.57 | 7.28 | 0.48 |
| **LMD** | Entorhinal IF | -0.69 | 9.26 | 0.43 |
| **CVLT- SFR** | Entorhinal IF  Arcuate ND | -0.45  0.34 | 11.47 | 0.56 |
| **CVLT- LFR** | Entorhinal IF  Arcuate ND | -0.58  0.28 | 16.63 | 0.65 |
| **CERAD - DR** | Entorhinal IF  IFOF ND  Hippocampal IF  Uncinate ND | -0.44  0.60  -0.30  -0.29 | 15.64 | 0.70 |
| **A. DTI Models** |  |  |  |  |
| **DRS** | IFOF FA  Hippocampal MD | 0.52  -0.32 | 11.51 | 0.54 |
| **LMI** | Entorhinal MD  Arcuate FA | -0.42  0.33 | 5.43 | 0.36 |
| **LMD** | Entorhinal MD  Arcuate FA | -0.50  0.30 | 7.09 | 0.42 |
| **CVLT- SFR** | Hippocampal MD  Arcuate FA | -0.48  0.29 | 10.25 | 0.53 |
| **CVLT- LFR** | Entorhinal MD  IFOF FA | -0.45  0.31 | 11.66 | 0.56 |
| **CERAD - DR** | IFOF FA  Entorhinal MD  Fornix FA | 0.42  -0.37  0.27 | 14.18 | 0.64 |

LMI, Logical Memory - immediate; LMD, Logical Memory – delayed; CVLT-SFR, California Verbal Learning Test – short delay free recall; CVLT-LFR, California Verbal Learning Test – long delay free recall; CERAD-DR, Consortium to Establish a Registry for Alzheimer’s Disease – delayed recall

* All regression models significant at *p* < 0.001
